# Holding momentum: a grounded theory study of strategies for sustaining living at home in older persons

**DOI:** 10.1080/17482631.2019.1658333

**Published:** 2019-08-27

**Authors:** Deborah Hatcher, Esther Chang, Virginia Schmied, Sandra Garrido

**Affiliations:** a School of Nursing and Midwifery, Western Sydney University, Richmond, Australia; b MARCS Institute for Brain, Behaviour & Development, Western Sydney University, Richmond, Australia

**Keywords:** Ageing, ageing in place, healthy ageing, home, independence, successful ageing

## Abstract

**Purpose**: Government strategies are putting increasing emphasis on sustaining the capacity of older persons to continue living independently in their own homes to ease strain on aged care services. The aim of this study was to understand the experiences and strategies that older people utilize to remain living at home from their own perspective.

**Methods**: A grounded theory methodology was used to explore the actions and strategies used by persons over the age of 65 to enable them to remain living in their own homes. Data were collected from 21 women and men in three focus group discussions and 10 in-depth semi-structured interviews.

**Results**: The data revealed that the central process participants used to hold momentum and sustain living at home involves a circular process in which older people acknowledge change and make ongoing evaluations and decisions about ageing at home.

**Conclusion**: These findings have implications for informing policy and service provision by identifying appropriate resources and services to promote successful ageing at home.

Today there are more than 617 million people aged 65 and over in the world, with the global population of people 80 and older likely to triple between the years 2015 and 2050 (He, Goodkind, & Kowal, 2016). Increased longevity and declining birth rates have contributed to this rise. However, while people are living longer they are often living with complex health needs in their later years. As the proportion of older people to those of working age increases, pressure is put on governments to provide adequate healthcare, pensions, transportation and housing. Institutional care for the aged is a major driver of increased costs to governments (Colombo, Llena-Nozal, Mercier, & Tjadens, 2011; Kok, Berden, & Sadiraj, 2015). Thus supporting older people to continue living independently in their own homes for as long as possible has become a priority for policymakers in many countries such as Australia and the UK (Australian Government Department of Health, 2018; Government Office for Science, 2016).

While there can be advantages to relocating to aged care accommodation especially in situations of deteriorating physical or cognitive health, research shows that quality of life is sometimes better when older people stay at home (Sixsmith et al., 2014). Some studies show that relocation to care accommodation can be associated with stress (Van der Pers, Kibele, & Mulder, 2015), loss of independence (Björk et al., 2016) and accelerated cognitive and physical decline (Harmand et al., 2014) while continued independence can enable older people to maintain wellbeing even in the face of illness and disability (Zeitler & Buys, 2015). On the other hand, living at home can assist older people to retain autonomy and control over the details of daily living (Van Dijk, Cramm, Van Exel, & Nieboer, 2015).

However, as people age, the increasing challenge of managing the activities of everyday living impact on the older person’s capacity to remain at home. For example, loss of mobility can restrict an individual’s ability to access community amenities such as doctor’s surgeries and supermarkets, and to participate in outdoor and social activities (Rantakokko et al., 2016). In Australia, support from family and friends is supplemented by a community care system while provides families with additional services such as home modifications or assistance with home maintenance to enable older individuals to remain living at home. In 2017–2018 more than 783,000 Australians were receiving home care under the Commonwealth Home Care Support Programme (Australian Government Department of Health, 2019). For people who can no longer live at home, respite or permanent residential care is available.

A number of concepts have become associated with the capacity to sustain living at home for older people, including “successful ageing”, “ageing well”, and “healthy ageing”, which relate to maximizing the quality of life and wellbeing of people in later life. However, older persons are not a homogeneous group (Van Dijk et al., 2015), and the health and social needs of individuals differ. Despite this, the perspectives of older persons who are managing to remain in their homes have received relatively little attention in the literature. There is limited research exploring how living at home affects health and the processes of daily life (Gillsjö, Schwatz-Barcott, & Bergh, 2013), and only a small amount of research exists on the perceptions and use of support by older persons themselves (Dawson, Bowes, Kelly, Velze, & Ward, 2015).

One early study in Australia by Day (1985) explored the reliance of older people on family support when no longer able to manage at home. Other studies have looked at housing adaptations to enhance self-care (Lubben & Damron-Rodriguez, 2003), home hazards (Gitlin, 2003), or how individuals connect emotionally to the physical space in which they reside (Bigonnesse, Beaulieu, & Garon, 2014). In addition, some studies have focused on community participation and relationships with other people in the locale (Aminzadeh, Dalziel, Molnar, & Garcia, 2010; Cristoforetti, Gennai, & Rodeschini, 2011). Other studies have looked at the benefits of particular activities that older people engage in such as outdoor adventure experiences (Boyes, 2013), life-long learning courses (Narushima, Liu, & Diestelkamp, 2018), and other leisure activities (Pereira & Stagnitti, 2008). However, this research does not explain how older persons make use of their own personal capacities and resources to sustain their capacity to live at home.

A limited number of studies have focused on the personal strategies employed by the older persons themselves. One cross-sectional survey study found that wellbeing in old age was correlated with self-management abilities (Cramm et al., 2013), confirming the importance of understanding individual strategies for managing age-related changes more closely. Stones and Gullifer (2016) examined the lived experience of 23 adults over the age of 85 years to better understand the adaptive strategies they employed to cope with the difficulties associated with very old age. Their study found that a number of historical, cultural and environmental factors had shaped the way individuals managed their day-to-day lives both psychologically and practically. However, further research is required to further eludicate the strategies that older people may use at various stages of the ageing process, and their approaches to managing age-related changes.

The current study aimed to add to an understanding of the phenomenon of older people living at home from their own perspective, by exploring their experiences and their understanding of the strategies they utilize to remain living at home.

## Method

After obtaining ethics approval from the Western Sydney University Human Ethics Committee, data was collected by means of focus groups and interviews, drawing on principles of grounded theory to facilitate theory development around the strategies older people use to remain living at home (Borbasi, 2008; Strauss & Corbin, 1990). This methodology has been widely used in the social sciences (Ormston, Spencer, Barnard, & Snape, 2014) and across nursing and midwifery disciplines (Brunstad & Hjälmhult, 2014; Jackson, 2014).

### Participants

Participants were included if they were (i) over the age of 65 years, (ii) English speaking, and (iii) had been living at home for at least 12 months before the commencement of the study, and (iv) consented to participate in the study. Participants were recruited from among regular attendees at classes held in a local government Seniors Centre in Western Sydney. The classes included line dancing, exercise and indoor bowls. The first author attended these classes on one occasion in order to speak about the study. There were no participants in attendance at these classes who were cognitively impaired and could not consent to participate. Pseudonyms are used to protect participant confidentiality.

Of the 21 participants in total, seven were married, 12 widowed, one never married and one divorced. Twelve participants lived in a house, six in a unit and the other three in a town house, retirement village and a duplex. The length of time participants had lived in their current dwelling ranged from 2.5–74 years. Almost all participants had lived in their current homes for at least 10 years and some had lived there for more than 40 years. The household composition included 12 living alone, five living with a partner, two with their children, and two with a partner and children (see Table I).

**Table I.** Participant profile.

Pseudonym	Gender	Age	Dwelling type	Marital Status	Education	Household composition
Maree	F	81	House	Widow	Primary	Lives alone
Mary	F	68	Unit*	Widow	Secondary	Lives with children
Tim	M	75	Duplex**	Divorced	Secondary	Lives alone
Ellen	F	73	House	Married	Secondary	Lives with husband
Peggy	F	78	House	Widow	Primary	Lives alone
Laura	F	66	House	Married	Secondary	Lives with husband & children
Tom	M	68	House	Married	Technical college	Lives with wife & children
Mark	M	66	House	Widow	Secondary	Lives with grandson
Jane	F	69	House	Widow	Tertiary	Lives alone
Kathy	F	72	House	Widow	Secondary	Lives alone
Sally	F	89	Unit*	Widow	Secondary	Lives alone
Sarah	F	82	House	Never married	Primary	Lives alone
Anne	F	79	Unit	Widow	Secondary	Lives alone
Irene	F	86	House	Widow	Secondary	Lives alone
Rose	F	88	House	Widow	Secondary	Lives alone
Alice	F	76	Unit	Married	Primary	Lives with husband
Frank	M	78	Town house	Married	Tertiary	Lives with wife
Dan	M	72	Retirement village	Married	Technical college	Lives with wife
David	M	66	Unit	Married	Tertiary	Lives with wife
John	M	97	Unit	Widow	Primary	Lives alone
Marjorie	F	78	House	Widow	Primary	Lives alone

*Participant moved to retirement village before end of study

**Participant moved to hostel before end of study

## Data collection

Data were collected via focus groups in the first phase followed by 10 individual interviews, following the model of Lambert and Loiselle (2008). This allowed topics to be initially explored in a generalized way in the focus groups, with more individual perspectives being garnered through the interviews.

Although no risk to participants was expected from this study, the assistance of a social worker was enlisted to which participants would be referred if they became upset or distressed while talking about their past or present experiences. Throughout the focus groups and interviews when participants raised uncomfortable or sad topics such as the loss of a partner, family member or friend, they were asked if they would like to discontinue the interview and/or talk to the social worker. No participants requested discontinuation of the discussion or referral to the social worker.

### Focus groups

Purposive sampling was first used in recruiting participants for focus groups in order to provide a broad collective perspective of the topic. Three focus groups were conducted: one consisting of three males and seven females, a second consisting of six females, and a third consisting of four males. Same gender groups such as those in groups 2 and 3 were used because of differences in interaction styles that can occur based on the gender composition of the group (Manderson, Bennett, & Andajani-Sutjaho, 2006).

Focus groups were conducted by the first author for approximately one hour in a small quiet room at the Seniors’ centre free from distractions with participants seated in a circular fashion behind tables. Discussions were audio recorded. A topic guide (Table II) that had been developed based on the literature was used to prompt the discussions following Kreuger and Casey's (2000) recommendation.

**Table II.** Focus group discussion schedule.

General introduction by the researcher
“Introduce yourself and state where you live and why you like living there.”
“Tell me about the things that make it possible for you to remain living in your home in the community?”
Other areas to cover: Home Community Independence Health and Wellbeing Resources Social network Support

### Interviews

Recruitment for the interviews occurred at the conclusion of each focus group. In total, 17 out of 20 participants in the focus groups volunteered to be interviewed. Theoretical sampling was used to select nine of the 17 volunteers, based on the concepts emerging from the analysis of the focus group data. Data from each interview were collected, coded and analysed to determine where to next collect data. Thus decisions about sampling were made as the study progressed. The researchers were most interested in the processes used when changing conditions impacted on everyday living. Five of the people who volunteered were not interviewed because their circumstances had not changed greatly and therefore did not require adjustments. Three of the volunteers passed away before they could be interviewed. The nine participants selected reflected a range of personal circumstances, health, and strategies used to sustain living at home. This allowed for maximum exploration of the processes used by the older persons. One female participant was interviewed twice due to changing circumstances over time.

In the first five interviews, four females over the age of 75 living alone, and one male living with a family member were chosen, as focus group data showed these participants had already put in place a number of processes to enable continued living at home. Participants for the other five interviews were chosen because their individual circumstances had changed significantly and become more complex due to deteriorating health. These five participants had either moved out of their home into alternative accommodation or were now living at home with higher levels of support.

Two participants were interviewed by the first author in their own homes, one in a hostel, and another over the phone at the retirement village. The remaining interviews were conducted at the Seniors Centre. All interviews were audio recorded. Interviews were conducted using an open-ended, semi-structured interview guide (Schneider, Whitehead, LoBiondo-Wood, & Haber, 2016), to enable the researcher to ensure coverage of essential topics while still allowing the interview to be largely participant directed (see Appendix).

Consistent with grounded theory, data collection and analysis proceeded interatively, with the emerging categories informing subsequent data collection. While a general approach was taken initially in the focus groups, concepts developed in analysis of focus group data informed development of the interview guide. Questions became more focused as the interviews progressed as patterns in the data became clearer (see Appendix). Data were collected over 24 months, including 16 weeks to conduct and analyse focus groups data. Data collection ceased when theoretical saturation was reached (Glaser & Strauss, 1967), i.e., when all categories including the central category were developed and no new categories relating to the central process could be developed by collecting more data.

### Data analysis

A process of microanalysis was first conducted by open coding of audio transcripts line by line (Strauss & Corbin, 2008). Initial codes were in-vivo, i.e., in the words of the participants themselves (Charmaz, 2006; Liamputtong, 2009). Later in analysis, axial coding was used to create categories by grouping similar concepts and exploring relationships between codes. The paradigm model of Strauss and Corbin (2008) was used. This model assists in the identification of whether the concepts and categories identified through the analysis represent the central phenomenon or are casual conditions, intervening conditions, context, action/interaction strategies or the consequences of the strategies. A template consisting of columns using these six headings within the framework was used. In processes of constant comparison, data were scrutinized alongside previous data to determine similarities and differences. Throughout this process diagrams were created and reflective memos were written to assist with theory development. In a final level of analysis, selective coding was used to integrate data categories in formation and refinement of an overarching theory (Grbich, 2007). As suggested by Strauss and Corbin (2008) a storyline was written to explain the central category and process, and the relationship of all categories in the paradigm model. In this way a central category, process and theory was developed.

## 

The data revealed a number of actions that older persons employ to fulfil their goal of remaining in their home, which contributed to the development of an overarching theory of *holding momentum*. These strategies fell into three major categories: *maintaining autonomy, protecting self, and connecting with others* (Table III). Participants also revealed interesting information about the entry point into the process of remaining living at home, or the point at which they began to take active steps to achieve this.

**Table III.** Major categories and sub-categories.

Maintaining Autonomy	Protecting Self	Connecting with Others
Drawing on inner self	Keeping well	Being there for each other
Shaping own approach	Staying safe	Positioning self

### Entry point

The commencement of deliberate strategies to sustain living at home often occurs when the older persons become aware of a threat to their ability to remain living there. It is these changes that are triggers for the *entry point* into the processes to stay living at home. The *entry point* was described as when something happened that made the person realize they had to be active in their desire to stay at home. For some in this study it was a major incident or sudden health crisis. For example, Rose described collapsing when travelling: “I go by train and the last time I was up there and I came back, I collapsed on (-) station … so the family has now said I’m not going on my own.” Mark described a sudden change in his health requiring surgery. Mark’s entry point was “after I got out of hospital … slowing down.”

For other participants a gradual change or deterioration in their health necessitated their entry point into the process of working to stay at home. Sarah said: “I was pretty good up ‘till … I would have been about 84, I started to find myself getting slower.” Tim described his entry point as the awareness of having to increase the effort required to get his everyday tasks done: “A bit harder to walk around. Up hill, more of an effort to walk up the street and then trying to get three meals a day, and the shopping.”

### Maintaining autonomy

Once the older persons in this study had entered the process of working to continue living at home, they worked hard to *maintain autonomy*. This category demonstrates how the older persons utilize their inner strengths and life experiences, and shape and modify their lifestyles to stay living at home. *Drawing on inner self* by using resolve and learning from past experiences, and *shaping own approach* through creating rules and making compromises, are the two subcategories that were combined to explain the complex action *maintaining autonomy* (Table IV).

**Table IV.** Subcategories and actions for major category: Maintaining autonomy.

Major category: *Maintaining autonomy*		
Subcategory	*Drawing on inner self*	*Shaping own approach*
Actions	Utilizing resolve	Creating own rules
	Depending on experiences	Making compromises

#### Drawing on inner self

The participants in this study all described using their resolve and learning from past experiences as actions enabling them to remain self-reliant and autonomous. Being autonomous means the older persons make their own decisions and take responsibility for their everyday living. Older persons spoke about relying on their strength and determination. Sally said: “You’ve just got to be strong and do it, that’s what I think.” The desire to maintain autonomy is a significant driver of participants in this study, while at the same time requiring determination. Sally stated: “As long as you’re doing everything for yourself. While you’re doing that you’ve got your independence.

Drawing on personal resolve was more important as persons experienced deteriorations in health. Anne said:

I think I’m more determined now than I was many years ago … you just get and do it, do the things that you think you can’t do, you just go get to and do them … . I do have a problem … high blood pressure … I’m still determined that I’m going to get there.”

Inner strength differs from individual to individual. For example, Irene described herself as “hard … tough … a tough old bird.” Anne similarly said, “If I know I’ve got to do something I just do it.”

In addition to utilizing their resolve, the older persons rely on learning from their previous experiences to remain autonomous. Peggy said that what she “learned to manage in earlier years of life is valuable for the later years.” Participants also draw on the knowledge and skills they had gained from their parents, partner and others. Jane for example, said: “The fact that I had a husband who refused to do absolutely anything … turned out to be helpful because when I was then alone I had to do everything for myself.” Similarly, Kathy said: “When my husband died I still had three children at school, so I have learned to be independent.”

Seeing other’s misfortunes also provides insight. Frank described how this was the catalyst for his decision to move into a more suitable home in the retirement village. He stated: “I have seen so much hardship amongst older people living on their own. I have seen a lot of homes actually neglected because they had no other help.”

#### Shaping own approach

This action involved not allowing others to interfere with everyday living, making compromises and giving up some things to be able to continue to do others. Participants were aware that to remain autonomous they needed to stay in control and not allow others to make decisions for them. For example, Rose described how she refused when her daughter suggested she move into accommodation with her: “My daughter suggested that I sell up and build a granny flat in their back … I said no I am quite happy in my own home.” Through undertaking the planning and organizing themselves, the older persons maintained their freedom and autonomy, preventing others from determining daily activities.

Determining their own pace also enables the older persons to manage their time and undertake daily activities within a desired timeframe. Sarah stated: “I take my time, I don’t hurry like I used to be able to hurry.” Sarah also described how she takes breaks in between domestic tasks: “I can only do a certain amount and I’ve got to stop … I rest more today than I used to.” Underlying these strategies is the ability to avoid getting concerned about completing work by pushing oneself at an unsafe pace. Thus, maintaining autonomy requires the older person to have an awareness of individual limitations, making decisions about when to compromise and being willing to do so. This action involved determining ways of negotiating the completion of tasks at home and in the community. Changing modes of transportation was one such adjustment to be made. As Tom said, “We used to drive everywhere but we are using more public transport now than before.”

Others learned how to do domestic tasks differently from what they were used to. For example, John adjusted his routine to shop more frequently so that he could continue to do the shopping himself: “I do a bit every week because you get a couple of big bags that are heavy to carry home.” Marjorie learned to shower at her daughter’s house rather than at home: “ … because she’s got a walk-in shower … at my place you’ve got to get into the bath … It’s a big bath … deep … I can’t always get my leg over the edge of the bath.”

### Protecting self

This category described the actions older persons used to maintain wellness to stay living in their homes. The two subcategories *keeping well* and *staying safe* described the complex action (Table V). Having good health for their age and the ability to keep active were essential features facilitating living at home. In addition, living in a home that was safe, protected the older persons from risks in the home and harm from the community.

**Table V.** Subcategories and actions of major category: Protecting self.

Major category: *Protecting self*		
Subcategory	*Keeping well*	*Staying safe*
Actions	Living healthily	Attending to risk
	Relying on health professionals	Making the home safe

#### Keeping well

One of the most common actions relied on by the older persons for *keeping well* was *living healthily*. Living healthily was described as adhering to good health practices including having good nutrition, avoiding unhealthy habits, keeping busy and occupied, and keeping mentally and physically active by engaging in activities in the community. For example, Irene described the way she maintains health through nutrition: “I’ve never had these quick meals much and I mostly cook my own fresh vegetables I get.”

Other participants looked after their health by avoiding unhealthy habits. Rose said, for example: “I don’t smoke, drink or gamble.” Some engaged in regular exercise to help them stay physically well. Examples included gentle exercise, weight bearing activities and recreational activities such as walking and playing bowls. For example as Kathy said, “I get out because I have got to keep my bones strong. I go walking … I am always on the go.”

Not keeping active was viewed as unhealthy. John commented, “I go out quite a lot because I do not like sitting around looking at myself and doing nothing because that does not do anybody, young or old, doesn’t do them any good.” Attending the seniors’ centre was a part of this strategy. Mary’s comment reflects this when she said: “The major interest of all this particular group is looking after our health by doing activities.” The older persons also described maintaining mental stimulation as an important factor in maintaining health. For example Anne said, “If you go out you meet people and you can talk to people and that keeps your brain active.” Ellen stated: “When we retired we decided we had to find something to really keep our brain active and something we could do together … We go out at least once a week playing bridge and it is beaut.”

The other main action for keeping well was to rely on health professionals. This involved maintaining a relationship with their general practitioner (GP). As Anne said, “I have blood pressure tablets and the doctor keeps a check on it.” The older persons in this study trusted their GP to provide advice, prescribe medications when necessary, offer appropriate testing and referrals for testing, and make home visits when required. Irene stated her doctor’s advice saying, “The doctors told me some years ago I should rest a lot more than I do … He said work a bit if you’ve got to but rest a bit too.”

Securing a doctor who makes home visits was another strategy considered essential, to assist with health issues when the older persons were unable to get to the surgery due to illness or lack of transport. Anne said, “It’s a big thing … they’ll always come if you want them to come to your home.” Marjorie similarly stated, “I just ring up and he comes. When I need him he’s there.”

A few older persons also mentioned using a physiotherapist. Maree, for example, said:

I walk every day and I have a physio that comes and gives me exercises so I’m doing all the right things to try and get myself, in the hope one day I’ll be able to do what I used to be able to do.

#### Staying safe

The older persons also took safety precautions, staying aware of risks in and out of the home and making changes to improve safety. For example, Anne said: “When I go up and down the stairs I’m always careful … I just go very slow, one step at a time … Hang on to the rail.” Using a walking stick or frame as a physical aid to minimize the risk of fall was another strategy utilized. For example, Mark said: “I was walking with a stick when I got home … Even down home nowadays I use the stick half the time … I get it out and just use that for balance.”

Others used technological services to attend to risk. For example, Frank used a service by the Australian Red Cross called Telecross that involves receiving a daily phone call to check on them, “so a person is not without help for more than 24 hours.” Maree similarly used VitalCall, a water resistant pendant that has an emergency call button that is worn by the older person. Maree said: “I know if I get sick I can press that and they are always there … . There is always someone there and that is a must when you are on your own.” Others had friends or neighbours on speed dial that they could call in an emergency.

For some, staying safe involved being aware of dangers outside the home. For example, Anne preferred living in an upstairs unit to minimize the risk of intruders: “I like living upstairs because I think you are safer upstairs.” They kept their doors and windows locked at all times. Rose said: “I always keep the security door locked in the daytime.” Others avoided going out at night, such as Irene, who said: “I always like to be home before dark … I just don’t go out at night … I make sure I get the bin out of a night before dark.”

Part of protecting self was transforming their home into a secure environment. Some older persons utilized an Occupational Therapist (OT) to assist them by assessing their needs for mobility and facilitating changes in their home. This often involved modifying the home environment by removing existing structures such as baths or steps that were considered unsafe, or making other changes as recommended by the OT. As Tim said:

I had a bath taken out before I moved in … so it is safe. It’s always a risk stepping into a bath no matter how careful you are, mats or anything at all, you can go for a very bad fall.

Jane similarly described: “When my sight went, I had great difficulties … the fellow put railing by the steps, back steps a sensor light so that I had light when I came home at night.”

### Connecting with others

The older persons in this study employed specific actions to form relationships with others that helped them to stay living at home. These formed two subcategories in the data: *being there for each other* by ensuring that others are available if needed, and *positioning self* to secure friendships and companionship and ensure formal and informal assistance is available when required (Table VI).

**Table VI.** Subcategories and actions for the category: Connecting with others.

Major category: *Connecting with others*		
Subcategory	*Being there for each other*	*Positioning self*
Actions	Having others there	Sharing with others
	Helping each other out	Outsourcing work

#### Being there for each other

Ensuring that others were around and present in their lives meant having support that was readily available. Ellen described how having her husband’s support meant that she could continue living at home:

If anything happened to him I couldn’t cope here by myself … Because of my eyesight he does the shopping and he reads the mail and there’s all sorts of things. He pays the bills.

Having others there also provided a sense of security and safety. As Rose said about having her family to call on: “You feel safe that somebody is there.” Ellen similarly talked about her adult children, saying: “They’re always at the end of the phone if you need them … We try not to impose too much of course. They’ve got their own lives … but we know that they’re there if we need them.”

The older persons took action to establish an informal system of support that was readily available through their family, friends, neighbours and others in their local community. They worked hard to stay connected with people in the community, to help each other and maintain a level of reciprocal support. Sally stated: “You help people and you get it back too. I have done a lot of that in my life.” Similarly Anne said: “If you have a good neighbour it makes a big difference. You could depend on your neighbour and she can depend on you.”

As well as having family and neighbours there to provide support, the older persons all described attending the senior centre regularly to ensure they have people around them for support. When speaking about the other older people attending the centre, Anne said: “You can always depend on somebody. If you really want anything there’s always somebody you can depend on.”

The older persons identified a major constraint on accessing support and staying connected as living alone. Jane described living without support when she said: “When you can’t drive and you haven’t got anybody to take you anywhere, that gets very difficult. People often leave their homes because they become too isolated.” For others, changes in the community with neighbours or friends moving away or dying impacted their ability to stay connected. As Rose said: “Most of them [neighbours] are gone … people on the corner, they’re all right … all I do is just say hello and how are you and that sort of thing.”

#### Positioning self

These strategies provided the older persons with friendships and companionship and the ability to outsource work when necessary. Communicating and socializing with others enabled the older persons to continue to feel connected. Anne described this as being “determined that you’re not going to sit and worry all day but to just go out and meet other people.” David described how communicating and socializing with others was particularly important. He said: “Even a phone call is really socialization … Communication is very important in old age.”

For many people, the seniors centre provided a place to socialize with others and sustain friendships. Irene said: “Coming to the centre is … the only social one outside of my sons that I see now.” Regularly attending enabled the older persons to feel part of a group. Anne said: “I spend my time with Senior Citizens two days a week and that keeps me occupied. You meet friends and you have friends when you go to these places.”

Staying connected with the community also enabled the older persons to take care of their daily needs by outsourcing. For example, Anne said: “I’ve this shop, it’s not far, it’s a small shop, an IGA shop. I just ring up and the girl gets the things ready and he brings them when he closes the shop.” To outsource work it was essential to learn about available services and how they could be obtained. As David said, “What a lot of people don’t know, who are at home on their own is that they should ring the council to see what is available … There are a lot of voluntary societies who can help you.” Recognizing the need to outsource and finding ways to do so, was essential to remain living at home.

### Holding momentum: theory development

**Figure 1.**
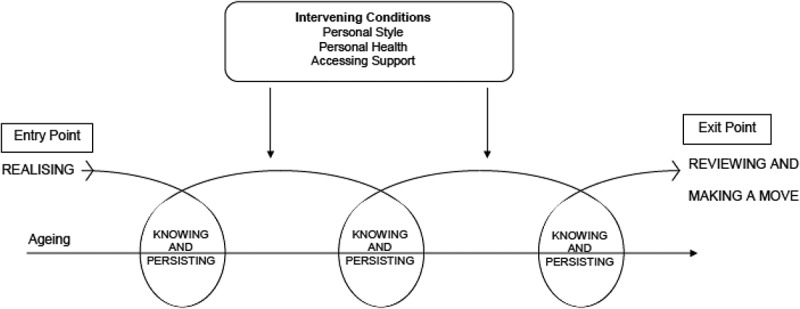
Model of holding momentum.

“Holding momentum” is the central process that links all categories and the strategies the older persons in this study used to sustain living at home. It is a process of continual decision making, re-evaluating and making new decisions about ageing at home (Figure 1). Although there were differences in the way the older persons individually engaged in the process and the length of time they spent in the process, when “holding momentum”, the older persons recognize personal and community change and make decisions to undertake the actions and interactions necessary to facilitate living at home despite these changes.

To successfully hold momentum, the older persons maintained continuous cyclical processes to mark time or “tread water” and stay independent. It is not a dynamic process because there is limited forward progression to a different state, but rather processes of working hard to stay in the same place. As depicted in Figure 1 these processes and actions commence at the time the older person *realizes* a need to enter the process and continue until they reach an *exit point* and make the decision to move to alternative accommodation. The older persons move through continual cycles characterized by *knowing and persisting*. Each cycle represents the strategies used and the confirmation of the decision to remain at home before moving into another cycle. Each time the older persons enter a new cycle they are required to draw on additional personal and community resources to sustain their living at home.

The older persons manage this process themselves, recognizing their own personal resources, strengths and limitations. They determine when to undertake tasks themselves or to engage the assistance of others. They have built a lifetime of community resources including friends, neighbours, family and health practitioners and have developed the ability to draw on the expertise and support of these resources when needed. Older persons with limited community resources draw heavily on their own personal resources and rely more on connections through groups such as the community centre.

The strategies of *maintaining autonomy, protecting self* and *connecting with others* are implemented in response to intervening conditions which are positioned above the cycles in the model as they continually impact upon them. The facilitating conditions of *personal strength, good health* and *access to support* assist the older persons to persist in the process whereas the conversely constraining conditions of *poor health* and *lack of support* impose the need for further changes. The characteristic *reviewing and making a move* is positioned at the end of the process within the model as it represents an evaluation of the situation and includes the *exit point* at which point the older persons may make the decision to move into alternative accommodation.

## Discussion

Many older persons want to remain living at home but have to face the changes that ageing brings. This study, in exploring the experiences of 21 people over the age of 65 years living independently in the community, identified the processes and strategies they used to manage age-related changes and remain living at home. Specifically, the study informed the development of a substantive grounded theory of older persons sustaining living at home. The findings suggest that to sustain their living at home, older persons require insight into the changes occurring and consciously draw on their individual resources, implementing a range of actions and strategies, including *maintaining autonomy, protecting self*, and *connecting with others.*

Some participants appeared to have greater inner resources than others. Their motivation and determination can be equated with the concept of resilience, which in turn is closely related to successful ageing (Pruchno, Heid, & Genderson, 2015). Learning from past experiences was also important in the current study. Similarly, previous studies have shown that learning from past experiences is important for ongoing problem-solving skills and the ability to manage health issues. For example, a systematic review by Mamykina, Smaldone, and Bakken (2015) demonstrated that being able to make connections between new observations and past experiences is important to self-management of long-term health conditions such as diabetes.

Whilst it is acknowledged that routines are highly important, the findings of this study illuminated the importance of insight into how and when to adjust or abandon routines in order to hold momentum and stay living at home. The participants in the current study made adjustments when routines became too demanding due to deteriorating health. Other research has confirmed the importance of adapting to changes in functioning to reserve energy (Dunér & Nordström, 2010). Participants also utilized numerous strategies to remain healthy. In particular, keeping mentally active and keeping physically strong were main concerns. This finding corresponds to other studies that have identified healthy eating and keeping active as major behaviours for ageing well (Michel, Cruz-Jentoft, & Cederholm, 2015). Similarly, other research has found that attending clubs or aged centres can prevent deterioration in cognitive function, by keeping individuals mentally and socially active (Shankar, Rafnsson, & Steptoe, 2015).

Negotiating and maintaining relationships with others in the community was another key strategy used by the older persons in this study. This included relationships with health professionals, family, friends and neighbours and others at the centre. The study found it was essential to secure relationships with others to have support and assistance when needed for immediate and future security, and to avoid isolation and loneliness. Other research has confirmed the importance of meaningful relationships (Nosraty, Jylhä, Taittila, & Lumme-Sandt, 2015) and shown how social networks can prevent declines in functional ability and improve quality of life (Kuiper et al., 2015).

It is important to note that in the current study these were negotiated relationships. Indeed, families provide a significant proportion of long-term care for older adults (Thomas & Applebaum, 2015). However, this often involves a substantial restructuring of family roles, and significant stress and adjustment for the families involved (Qualls, 2016). Relationships between the older person themselves, family, and government provided services must often be a process of careful consultation and negotiation, a challenging process where the aims of the parties may be conflicting (Dunér & Nordström, 2010). Future research is needed to further consider how these relationships are navigated and negotiated both when living at home and when older persons relocate to residential care services.

In the current study, decision-making was influenced by many factors that may be explained by the push-pull theory, a framework often used in relocation decision-making (Smetcoren, De Donder, Dury, & De Witte, 2017) . According to this theory, push factors are those that push or drive people out of a place so they make a decision to relocate, and pull factors are those that pull or draw people towards a place. In this study, the major push factors to exit the process of holding momentum were deteriorating health and lack of support. These findings concur with other studies which describe how the increasing challenges of everyday living and poor health can threaten independence and a sense of dignity (Granborm, Löfqvist, Horstmann, Haak, & Iwarsson, 2014; Lloyd et al., 2017).

In the current study, few participants experienced the “pull” of attraction to alternative accommodation in the form of less home maintenance or provision of care, despite the fact that previous research suggests that older persons tend to change to smaller housing and from owner-occupied to rented housing for these reasons (Abramsson & Andersson, 2016). In this study, participants were actively engaged in strategies that were designed to help them continue living at home. Despite this preference in many older persons to remain living at home, research findings about the relative benefits of home living and care accommodation is mixed. One study (Kok et al., 2015) showed that older persons over the age of 75 living in residential care were happier than those living at home. Another study (Rodriguez-Blazquez et al., 2012) showed that younger individuals in the aged sector (< 78 years) are happier at home, while older individuals are happier in residential care because of the greater support provided for loss of functionality and social support. Nevertheless, because of the reduced costs to governments of home living, policies can tend to focus on promoting home care (Kok et al., 2015). Additional research is needed to consider the relative benefits of each, as well as the processes used by older persons and their families in making decisions about living arrangements as age related challenges increase.

The findings of this study also demonstrate that the older persons personal strength, health, and access to support, and not just the sustainable environment, influence their capacity to make decisions and implement strategies to enable continued living at home. Despite many points of similarity, each participant’s experience of the process was individual, demonstrating the need for flexibility in policy and service provision. A comprehensive understanding of these issues and implications can assist policy makers, healthcare professionals and older persons themselves to sustain living at home.

## Conclusions

The current study was limited by the nature of the participant group. This group was a relatively healthy and active group, as evident from the context in which they were recruited. Future research should seek information from more divergent cases, specifically those from other cultures and different living arrangements. The methodology used also had some limitations. Focus groups can be useful for drawing out multiple perspectives. However, group dynamics can shape the perspectives expressed by participants. The fact that group members were known to each other can also influence how people communicate about potentially personal topics. While this limitation was overcome to a certain extent by the follow up interviews where participants could express personal perspectives individually, results should be read with this limitation in mind.

Nevertheless, this research contributes useful knowledge for policy makers and highlights the importance of knowledge from older persons themselves in designing policies about community care and service provision. The study illuminates the need for services that meet individual rather than population needs, and that build on the strengths of older persons. Because successfully holding momentum is largely a personal achievement, dependent on the older persons’ degree of resilience, the implications drawn from the study for people in this situation are centred on maintaining their health and independence. This strongly suggests that focusing on health and support is vital to maximizing the capacity of older persons to continue living at home.

This research also has significance for health service providers as it identifies existing gaps in health service provision. In particular, the study highlighted the need for older people to maintain control over the decision making process. Health service providers need to help older persons identify appropriate, accessible, and affordable resources, and promote ease of access to these resources. Furthermore, understanding factors affecting living at home will help health service workers develop strategies to support older persons. Through greater understanding of the process by all concerned, older persons in the community will enjoy longer, more happy and fulfiling experiences in the later years of their lives, staying in their homes which mean so much to them.

## Appendix Interview Schedule

**Interviews 1–5**

- What are the most rewarding aspects of living in your home?
- In what ways does your health enable you to live at home?
- What do you think are your personal attributes (qualities) that enable you to stay living at home?
- What are the things that make it possible for you to stay living there?
- How does being in a community assist you to live at home?
- What are some of the challenges of living in your home?
- Have you thought about what you will do in the future?
- When do you think you started to think about remaining living at home?
- What do you think would be a reason that you may not be able to stay living in your home?

**Interviews 6–10**

- What is it about your home that makes you want to stay living there?
- What are the things that have helped you to stay living there?
- What are the things that sometimes make it difficult for you to live there?
- Were there times when things happened and you had to make changes so that you could stay?
- Do you ever think about the future and what might make you move out?
- What are the attributes you have that help you stay at home?
- What are some things you do to stay at home?
- What support do you have and how often do you use it?
- What support do you give to others?
- What work do you get others to do for you?
- What are some of the negative things about living at home for you?
- What are some of the positive things about living at home for you?
